# Astrovirology: how viruses enhance our understanding of life in the Universe

**DOI:** 10.1017/s1473550423000058

**Published:** 2023-04-05

**Authors:** Gareth Trubl, Kenneth M. Stedman, Kathryn F. Bywaters, Emily E. Matula, Pacifica Sommers, Simon Roux, Nancy Merino, John Yin, Jason T. Kaelber, Aram Avila-Herrera, Peter Anto Johnson, John Christy Johnson, Schuyler Borges, Peter K. Weber, Jennifer Pett-Ridge, Penelope J. Boston

**Affiliations:** 1 Physical and Life Sciences Directorate, Lawrence Livermore National Laboratory, Livermore, CA, USA; 2 Center for Life in Extreme Environments, Department of Biology, Portland State University, Portland, OR, USA; 3 Honeybee Robotics, Altadena, CA, USA; 4 NASA Johnson Space Center, Houston, TX, USA; 5 University of Colorado Boulder, Boulder, CO, USA; 6 DOE Joint Genome Institute, Berkeley, CA, USA; 7 Chemical and Biological Engineering, Wisconsin Institute for Discovery, University of Wisconsin-Madison, Madison, WI, USA; 8 Institute for Quantitative Biomedicine, Rutgers, the State University of New Jersey, Piscataway, NJ, USA; 9 Computing Directorate, Lawrence Livermore National Laboratory, Livermore, CA, USA; 10 Faculty of Medicine & Dentistry, University of Alberta, Edmonton, AB, Canada; 11 Northern Arizona University, Flagstaff, AZ, USA; 12 Life & Environmental Sciences Department, University of California Merced, Merced, CA, USA; 13 NASA Ames Research Center, Moffett Field, CA, USA

**Keywords:** ecology, evolution, exobiology, life, origin, planetary protection, space

## Abstract

Viruses are the most numerically abundant biological entities on Earth. As ubiquitous replicators of molecular information and agents of community change, viruses have potent effects on the life on Earth, and may play a critical role in human spaceflight, for life-detection missions to other planetary bodies and planetary protection. However, major knowledge gaps constrain our understanding of the Earth’s virosphere: (1) the role viruses play in biogeochemical cycles, (2) the origin(s) of viruses and (3) the involvement of viruses in the evolution, distribution and persistence of life. As viruses are the only replicators that span all known types of nucleic acids, an expanded experimental and theoretical toolbox built for Earth’s viruses will be pivotal for detecting and understanding life on Earth and beyond. Only by filling in these knowledge and technical gaps we will obtain an inclusive assessment of how to distinguish and detect life on other planetary surfaces. Meanwhile, space exploration requires life-support systems for the needs of humans, plants and their microbial inhabitants. Viral effects on microbes and plants are essential for Earth’s biosphere and human health, but virus–host interactions in spaceflight are poorly understood. Viral relationships with their hosts respond to environmental changes in complex ways which are difficult to predict by extrapolating from Earth-based proxies. These relationships should be studied in space to fully understand how spaceflight will modulate viral impacts on human health and life-support systems, including microbiomes. In this review, we address key questions that must be examined to incorporate viruses into Earth system models, life-support systems and life detection. Tackling these questions will benefit our efforts to develop planetary protection protocols and further our understanding of viruses in astrobiology.

## Introduction

Viruses are numerically abundant and an integral part of life on Earth. However, there is very little known about viruses across many of Earth’s environments, including the many extreme habitats that serve as astrobiology analogues, when compared with the understanding of viruses relevant to human health. Even with respect to human health, relatively little is known about viral responses to the space environment beyond Earth’s atmosphere. Additionally, almost no research has been conducted to identify the impact of these viruses on the spacecraft systems used to sustain humans. Understanding viruses and virus–host interactions in space-relevant contexts will inform our grasp of their role(s) in both human spaceflight missions, including environmental control and life support systems (ECLSS), and in the search for life elsewhere. Here, we review viruses in diverse Earth environments, the very limited studies conducted on viruses in space and lay out a roadmap for future research. For a more extensive overview of environmental viruses and introduction to viruses, see Berliner et al. (2018); for a specific review on the roles of viruses in spaceflight affecting human health, see Pavletic et al. (2022); for a complementary review on the need to incorporate viruses more in astrobiology, see De La Higuera and Lázaro (2022).

Viruses are sometimes defined as ‘very small obligate intracellular parasites’ (Acheson, 2011), and more broadly as ‘entities whose genomes are elements of nucleic acids that replicate inside living cells using the cellular synthetic machinery and causing the synthesis of specialized elements that can transfer the viral genome to other cells’ (Luria et al., 1978). These ‘specialized elements’ or virions are crucial to the definition of viruses. Viruses contain genetic information that can be transferred on massive scales due to the very large numbers of virions on Earth.

**Figure 1.**
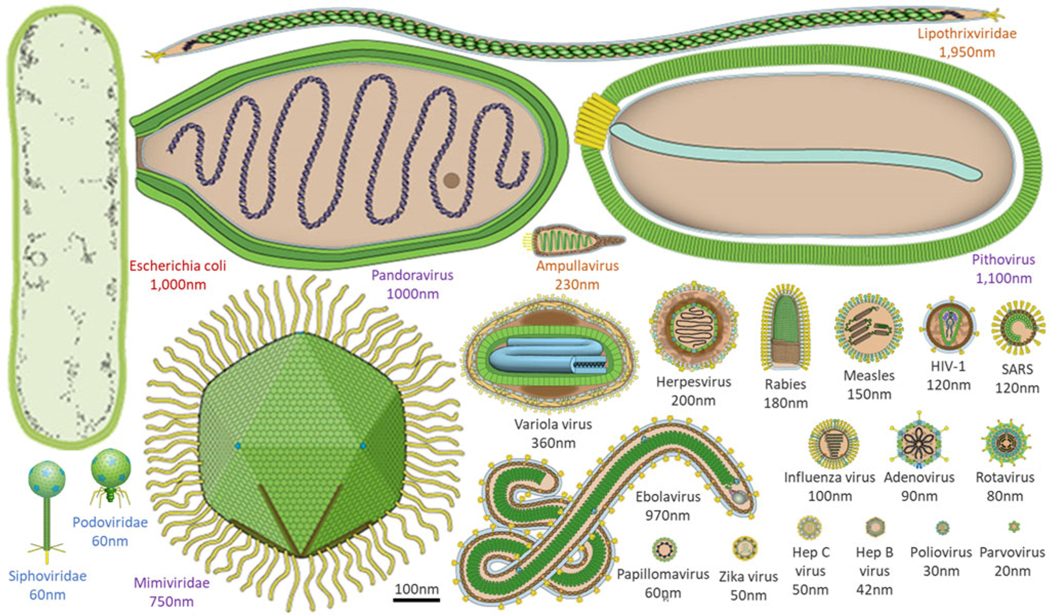
Morphological comparison of viruses, and a common bacterium at the same scale. Viral morphology and size are highly diverse, ranging from a few to thousands of nanometres. Here, an example bacterium (Escherichia coli, red text) is contrasted with example viruses that infect humans (black text), amoebae (purple text), archaea (orange text) and bacteria (blue text). Virus images adapted from ViralZone, Swiss Institute of Bioinformatics.

The sheer ubiquity of virions on Earth, with up to 10^31^ in the Earth’s oceans alone (Suttle, 2005; Mushegian, 2020), makes them an attractive target for the search for life on other worlds if one only knew what to search for! Viruses that infect bacteria and archaea typically range in size from tens to a few hundred nanometres, while viruses that infect eukaryotic organisms (e.g. amoebae or humans) can even range from tens to thousands of nanometres (Fig. 1). These so-called giant viruses can be larger than some bacteria or archaea and can even be infected by viruses themselves (Sommers et al., 2021). For example, virophages have been observed to infect giant viruses that are themselves infecting cellular organisms (e.g. amoebae). In these cases, the virophage infection co-opts the giant virus and may improve the condition of the host organism (Roux et al., 2017; Backstrom et al., 2019; Schulz et al., 2020). Viruses can also alter the genetic architecture, phenotypic characteristics, reproduction strategy, infection dynamics and evolution of nearby viruses, which in turn influences how viruses interact with their hosts and ultimately, ecosystems. This spectrum of virus behaviours across all domains of life is also reflected in the productivity of their infected host cells (i.e. modulation of host-cell metabolism and tens to thousands of progeny virus particles per cell) and timing of single-cycle infection (e.g. tens to thousands of minutes). Notably, the timing for one cycle of virus growth correlates with the doubling time of healthy host cells (Jin and Yin 2021).

The diverse morphology of viruses which extends beyond that typically found in cellular life (Fig. 1), and the presence of unique genes for the virus coat or capsid, have already been used as a model for other life: ‘capsid-encoding organisms’ (Forterre and Prangishvili, 2009). Virus genomes can be comprised of DNA or RNA, double-stranded or single-stranded, in multiple variations of each. Unlike cellular life, there are no universal viral genes to serve as a backbone upon which to build consistent evolutionary trees (Roux et al., 2019; Tisza et al., 2020). The diversity of both virus morphology and viral nucleic acids makes them difficult to search for comprehensively, but also drives expanded toolkits that could be employed in the search for life on other worlds and therefore deserves further investigation.

Virus-like entities may even be detectable on other worlds where life-like processes are only just evolving. Although humans have become explicitly aware of viruses and their effects only in the past few centuries (Loeffler and Frosch, 1897; Beijerinck, 1898), they were likely present as soon as – or even before – the first cell arose on Earth (Moelling and Broecker, 2019). Viral infections have played a key role in the evolution of life on Earth via the exchange of genes between viruses and their hosts, providing genetic diversity, killing dominant hosts thus maintaining balanced populations and possibly even inventing DNA itself (Forterre and Prangishvili, 2009; Enard et al., 2016). Viral genome remnants are present in most, if not all, cellular genomes, including in humans, where viruses play key roles in human physiology (Bannert and Kurth, 2004; Arneth, 2021). Further studies of the history of Earth’s viruses may highlight opportunities for detecting emergent life on other worlds.

On Earth, viruses have such a substantial impact that they drive major global biogeochemical cycles, and the same may be true on other worlds. The vast majority of marine viruses infect microbes (Suttle, 2005; Parikka et al., 2017), thus affecting many biological processes, including encoding photosynthesis genes that substantially contribute to atmospheric oxygen (O_2_), and help regulate global nutrient cycling (Greene and Reid, 2013). Further understanding of the persistence of virus particles and viral impacts on microbial cellular chemistry may therefore help illuminate biosignatures on distant planets, or signatures of past life in the geologic records of other worlds.

Targeting the search for biosignatures should focus on locations that are currently or once were within the environmental ranges compatible with the persistence and replication of information molecules. The more that research is conducted on viral persistence and replication in extreme environments, the more that remarkable feats of persistence and replication may be discovered (e.g. viruses thawed from permafrost could infect a modern-day version of their host; Legendre et al., 2014).

Viral persistence in and response to extreme conditions also has important implications for humans voyaging into space, as recently reviewed by Pavletić et al. (2022). However, in addition to viral impacts on human physiology via direct infection and impacts on the microbiome, viral infection of plants or microbes could impact critical life support systems on deep space missions, including algal bioreactors (Matula and Nabity, 2019). Conditions such as microgravity affect fluid dynamics with implications for bacterial biology (as reviewed in Diaz et al., 2019; Acres et al., 2021; Sharma and Curtis, 2022), and presumably also virus–host interactions, but this is still an underdeveloped field.

The diversity of unique viral qualities and their integral role in life on Earth compels us to better understand the potential roles of viruses and virus-like entities in both astrobiology and space biology. There are multiple critical knowledge gaps in our understanding of viruses in the space environment and their potential role in other hypothetical biospheres in our Solar System and beyond.

In this review, we outline what new virus research here on Earth should investigate to address key scientific questions in planetary science, space biology and astrobiology. These include:

1. What role(s) did viruses play in the origin and evolution of life on Earth?
2. What are the environmental limits to the preservation and propagation of virions?
3. What role(s) could viruses play in potential biospheres elsewhere, and how might those impacts be detectable as biosignatures?
4. Can the detection of viruses beyond Earth be interpreted as a sign of life?
5. How do artificial environments affect the human virome?
6. Can virions be fossilized or otherwise preserved in a recognizable state?

### Potential role(s) of viruses in the early stages of life

**Figure 2.**
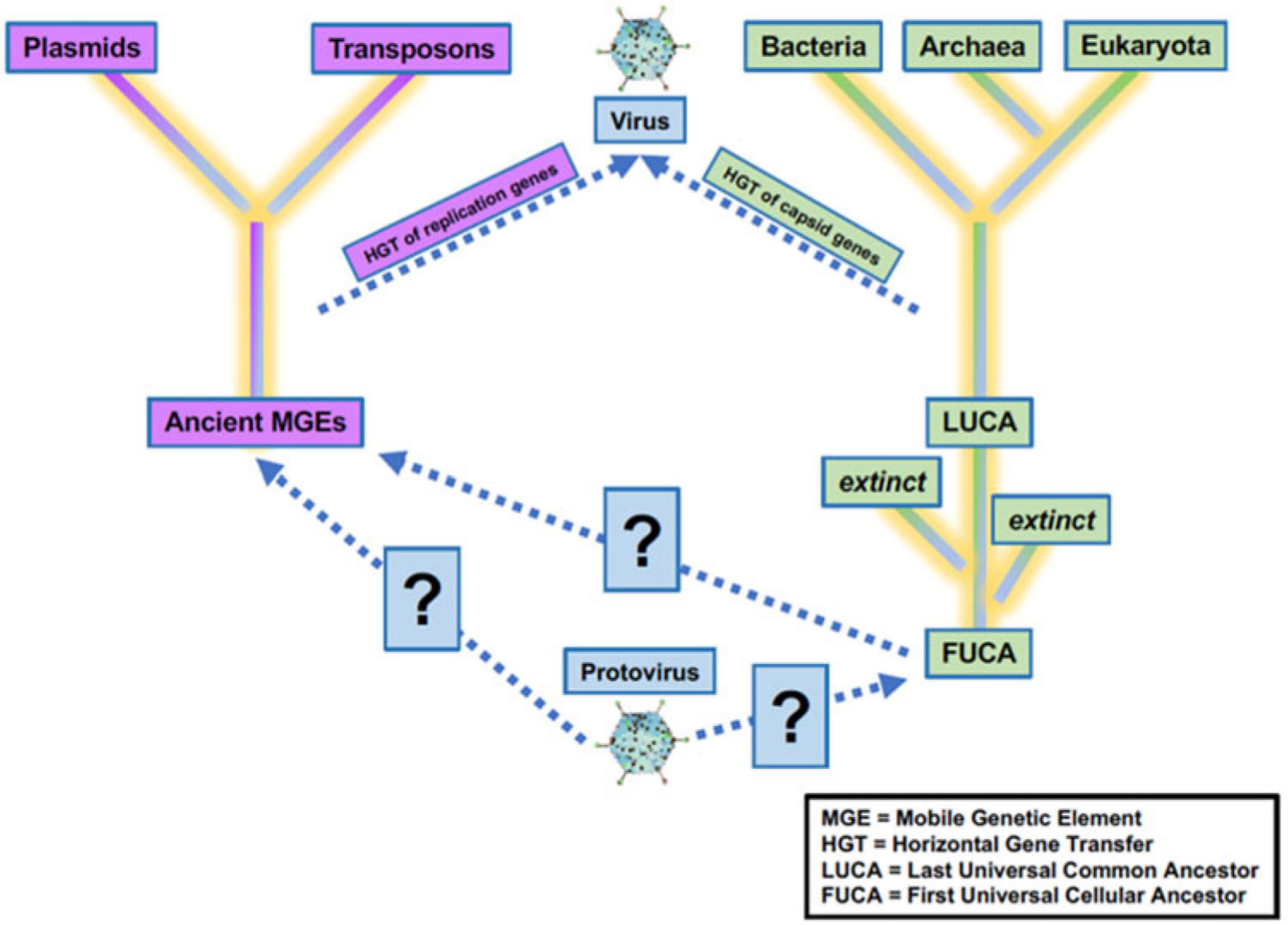
Uncertainty in the origin(s) and evolution of life on Earth. Viruses play a critical role in the evolution of life as we currently know them, and viruses or precursors of virus-like entities must be considered in origin experiments. Adapted from Harris and Hill (2021).

Increasing knowledge of the roles of viruses in the origins of life on Earth will benefit the search for life elsewhere. Viruses have and will continue to play a critical role in the evolution of life on Earth. Precursors of virus-like entities may even mark the beginning of life itself, making the virosphere potentially as old and significant as the cellular biosphere (Fig. 2; Janjic, 2018; Moelling and Broecker, 2019). Viral signatures may be pivotal in the search for life elsewhere and in understanding evolutionary mechanics of other biospheres. Theoretical models predict that (depending on the information-transfer properties of the system) parasitic replicators will emerge anywhere in the Universe that life-like processes evolve (Eigen, 1971; Bresch et al., 1980; Vignuzzi and López, 2019; Vlok et al., 2019). Indeed, even viruses themselves fall prey to parasitic replicators in the form of defective virus genomes and virophages that multiply at the expense of fully intact virus genomes (Perrault, 1981; Vignuzzi and López, 2019; Roux et al., 2023). The ‘RNA-world’ hypothesis characterizes the origin of life with self-replicating RNA, followed by ribonucleoproteins that later evolved into DNA and larger proteins (Müller et al., 2022). Viruses and virus-like replicators are the only known extant biological entities that contain all types of nucleic acid genomes (Table 1), including single-stranded and double-stranded RNA and DNA or mixtures thereof. RNAviruses serve as models for how RNA and ribonucleoproteins could have propagated via simple self-replicating RNA structures and ribozyme activity (Landweber et al., 1998; Koonin et al., 2006; Tyler, 2008; Durzyńska and Goździcka-Józefiak, 2015; Matsumura et al., 2016; Weinberg et al., 2019). Viruses may have helped support the transition from the early RNAworld to the current DNAworld (Forterre, 2006; Diemer and Stedman, 2012). Further, the recent acceptance of network-based clustering for virus taxonomy (Jang et al., 2019) in addition to incorporation of three-dimensional (3D) structures (and other viral characteristics) have allowed deep evolutionary inferences that have identified newly proposed phyla of RNA viruses which may be a previously missing link in the transition from the RNA–peptide world to the DNA–RNA–peptide world (Zayed et al., 2022). Further, beyond the origins of life, viruses played a role in the origins of the mitochondrion (Shutt and Gray, 2006) and of the placenta (Mi et al., 2000). Viruses may even have been critical in the evolution of eukaryotes and multicellularity (Forterre and Gaïa, 2016; Lee et al., 2018; Guglielmini et al., 2019). Overall, viruses appear to have played many roles in the origin and evolution of early life.

While uncovering the history of viruses is key to understanding the origin and evolution of life on Earth, an additional benefit is the development of new technologies that are potentially valuable for characterizing the diversity and history of non-terran life. For instance, comparative analysis of 3D atomic structures is a non-genomic method being used to interrogate virus origins that could also be applied to non-terran samples that may lack nucleic acids (Bamford et al., 2005). This method is employed to detect and classify viruses because there are no genes shared by all extant viruses (Roux et al., 2019; Tisza et al., 2020). In comparison, all organismal cells conserve some genomic features such as ribosomal RNA (Table 1). Thus, the search for life elsewhere can tap into such techniques. In particular, we propose the following future research directions.

#### Future directions

- Investigate evolutionary relationships between RNA viruses and ribozymes (ribonucleotide enzymes) to evaluate the potential role of viruses in an RNA world, and the transition to a DNA world.
- Create tools and methods to enable inferences about early life through comparative analysis of extant viral life.
- Reconstruct ancient events to illuminate the origin(s) of viruses on Earth, and their possible roles in the emergence of all life.
- Develop phylogenies that consider all biological entities to attempt to connect and better constrain the shared and divergent evolutionary histories of viruses and cellular organisms.

### Habitability, persistence and process limits of viruses and cellular life

Viruses have been known to remain active in a variety of harsh conditions, sometimes influencing the functioning and preservation of their hosts and sometimes reinfecting hosts after being preserved themselves yet still viable. Cellular life can also persist and operate under a variety of extreme conditions on Earth. However, the monitoring of virus–virus and virus–host activity together in extreme environments has been limited, despite these interactions being inherently different than those in milder environments. Therefore, fundamental concepts are not well understood about the types of environmental conditions and processes that are most conducive to the preservation and propagation of information molecules or molecular signatures of life. For example, understanding the upper and lower limits of viruses’ persistence and activity under a range of conditions would inform where we might find viruses and other life on both Earth, in space and on other worlds. Additionally, long-term monitoring of viruses in extreme environments could inform their ability to persist under similar conditions on another planetary body. Multiple analytical techniques could be used to better determine viral activity in both dormant and active host environments and to better characterize viruses and low-level host activity as well. While viral process limitations are currently unknown for a range of conditions, viruses persist and interact with their hosts under a wide range of extreme conditions on Earth (Gil et al., 2021), including extremes in temperature, pH, pressure and salinity. These conditions manifest in both natural and built environments, and viruses have been observed in a variety of settings (Table 2). Understanding the extreme ranges of conditions in which viruses and their hosts can survive informs the search for life on other worlds, the operational considerations for planetary protection and viral monitoring during human spaceflight.

In a variety of environmental settings, viruses can persist, maintain their integrity and even help preserve their hosts for extended periods of time. For example, viruses that adsorb to clays are protected from inactivation, thus enabling them to persist in soils for longer periods of time in the absence of a host (Bitton, 1975; Lance and Gerba, 1984; Lipson and Stotzky, 1985; Syngouna and Chrysikopoulos, 2010). Virus particles can also be preserved via silicification and can become reactivated (i.e. infectious once again) when the silica is removed (Laidler and Stedman 2010; Laidler et al., 2013). Further, upon thawing, viruses preserved in frozen permafrost for ~30 000 years were able to infect modern day versions of their hosts (Legendre et al., 2014). Viruses can also influence the preservation of their biological hosts. For example, viruses that infect microbial mats can cause or expedite the microbial mats’ fossilization into a stromatolite by (1) acting as a nucleation site for organomineralization, (2) altering microbial host metabolism to promote carbonate precipitation or (3) increasing the production of microbial extracellular substances (Pacton et al., 2014; White et al., 2021). In summary, viruses can function as key structures for preservation and propagation of biological information, especially in extreme environments. In this context, a better understanding of which environmental and molecular factors drive this long-term persistence will enhance future searches for virus-like elements outside of Earth.

Throughout extreme environmental conditions, viruses can also extend protection to their hosts. For instance, viruses can confer host heat tolerance (Márquez et al., 2007), and carry genes for sporulation that may drive their host to form an inactive spore, that is robust to unfavourable conditions (Trubl et al., 2018; Van Goethem et al., 2019; Pelusi et al., 2021; Travers et al., 2022). In another instance, viruses allow photosynthesis in cyanobacteria under desiccating conditions (Azua-Bustos et al., 2012). Harsh environments, such as polar regions or hydrothermal vents, have a high incidence of temperate viruses, which can reside within their microbial hosts (lysogeny) until conditions are favourable for viral replication (Anderson et al., 2014; Brum et al., 2016). While in this lysogenic state, viruses may express genes that alter their microbial host’s physiology and metabolism, thus increasing the Table 2. Example locations where viruses have been observed through isolation or genomic studies host cell’s ability to survive conditions in which resources are limited, including within sea ice, dry soil or acidic environments (Chen et al., 2005; Yu et al., 2015; Howard-Varona et al., 2017; Lee et al., 2021; Wu et al., 2021).

By integrating into hosts that are dormancy capable, viruses may be able to persist through conditions that are incompatible with activity then reactivate when conditions improve. Extreme examples of organisms regaining function after dormancy include: (1) cyanobacteria reviving after a 672 day exposure to space outside of the International Space Station (ISS; Laranjeiro et al., 2021), (2) cyanobacteria in Antarctica metabolizing within a week after rewetting following 20–30 years without stream flow (McKnight et al., 2007), (3) nematodes reviving from 30 000-year-old permafrost in Siberia (Shatilovich et al., 2018) and (4) rotifers in northeastern Siberia reactivating from 24 000-year-old permafrost (Shmakova et al., 2021). Microbes living under low-energy conditions in the South Pacific Gyre are claimed to have even retained their metabolic response after 101.5 million years (Morono et al., 2020). The diverse evidence of microbial and eukaryote dormancy noted above shows the potential for organisms to survive and even grow in space-like environments and planetary analogues. However, not much is known about any hitchhiking viruses, that have accompanied these long-persisting hosts, nor how they may have contributed to long host dormancies. Evaluating the persistence and reactivation of viruses in dormant hosts would hint at the plausibility of their presence in extreme non-terran environments.

The effects of viruses on extremophiles involve virus–host interactions that are fundamentally different relative to ideal environments (Dávila-Ramos et al., 2019). The monitoring of viral persistence and activity in a wide variety of extreme environment types may indicate how viruses survived during early Earth, and what is applicable to alien biospheres. We need expanded experimentation to determine end member limits of viral persistence and activity under conditions like aridity, humidity, low/high O_2_ content, trapping in amber or soil/sediments, etc. Scoping out the broad viral environmental envelope would point to where virus-like entities might be found on both Earth and other worlds.

We posit that similar virus–host interactions are likely on any planetary body where life exists, thus it is critical to expand our understanding of these interactions on Earth to enable the detection and identification of such phenomena during space missions. For example, it is now feasible to computationally integrate how functions encoded in virus genomes interact with the material and energy resources of their hosts, thereby predicting the timing and levels of virus growth (Yin and Redovich, 2018). Successfully predicting viral activity in human-support space environments would help assure astronaut health.

Viral activity in low-energy Earth environments can be explored through development of high sensitivity tracer approaches. Bulk tracer methods, such as nucleic acid stable isotope probing (SIP; Radajewski et al., 2000; Manefield et al., 2002), can provide detailed information about the level of stable isotope incorporation by microbes and have been applied to investigate virus activity in complex environments (Pasulka et al., 2018; Lee et al., 2021, 2022; Starr et al., 2021; Trubl et al., 2021). Bulk methods such as SIP integrate over relatively large sample mass (Nuccio et al., 2022), thus requiring more enriched substrate in complex systems. However, investigators have recently developed imaging mass spectrometry approaches that can detect viral replication at the single-particle level based on incorporation of rare stable carbon and nitrogen isotopes (Gates et al., 2018; Pasulka et al., 2018; Mayali et al., 2019). Because virion enrichment has been detected in individual particles using nanometre-scale secondary ion mass spectrometry (NanoSIMS), the approach is clearly equally viable for other very small samples. These studies have demonstrated sensitivity down to 100 nm-diameter virions. Further research is necessary to extend these methods to bacteriophage in the 50 nm-diameter range and experimentally apply them to extreme environmental and space-like conditions. Such powerful techniques could be used to evaluate the resilience of viruses to astrobiologically relevant conditions, thus we propose the following experimental directions.

#### Future directions

- Increase of environmental surveys and long-term monitoring of viral persistence and activity in extreme environments that can be used as analogues of planetary bodies and early life on Earth.
- Experimental work on the lower and upper limits to viral persistence and activity under a variety of environmental parameters.
- Evaluation of the persistence and activity of viruses in dormant and active hosts, combining techniques (e.g. SIP with metagenomics) to better characterize viruses and detect low-level host activity.

### Biogeochemical cycling and biosignature detection

Viruses play critical roles in biogeochemical cycles on Earth. Our understanding of viruses as major players in Earth’s biogeochemical cycles has been reshaped by the advent of metagenomic approaches that have enabled the study of uncultivated viruses (Kristensen et al., 2010; Roux et al., 2016, 2019). Marine virology has been particularly intensely studied, highlighting the potential for worlds with liquid oceans, such as Enceladus or Europa, to contain informational molecules such as virus genomes and proteins amongst their organic compounds (Postberg et al., 2018). On Earth, viruses have a huge impact on O_2_ concentrations by infecting the marine cyanobacteria that are responsible for ~25% of the O_2_ in Earth’s atmosphere. At any moment, about half of marine cyanobacteria are infected, which can lead to either a decrease in O_2_ production (i.e. cells lysed by viruses) or an increase in the efficiency of their O_2_ production (Sieradzki et al., 2019). We need an improved understanding of what controls that balance, and how can that be used in biogeochemical modelling. Other work has demonstrated the activity of viruses in cold to sub-freezing temperature soils (Trubl et al., 2021; Wu et al., 2022) and illustrates the potential for viruses in biogeochemical processes of cold terrestrial worlds, such as Mars. Terrestrial worlds, or at least partly rocky bodies, such as comets, may host ice-lidded cryoconite holes in their polar ice caps that could result in the presence of liquid water to support active life (Zawierucha et al., 2017). Could terrestrial worlds with thick mid-deck atmospheres, such as Venus, harbour life and virus-like entities? On Earth, we know that viruses exist within droplets in the atmosphere and can augment iron and sulphur metabolisms (Anantharaman et al., 2014; Bonnain et al., 2016; Roux et al., 2016; Dalcin Martins et al., 2018). Since Venus’s clouds contain sulphuric acid, viruses could be influencing biogeochemical processes in the planet’s atmosphere as well. More intimately studying the role of viruses in biogeochemical processes in a variety of Earth analogue environments could inform any potential role viruses might play in biogeochemical processes on other worlds.

The widespread and frequent detection of genes used by viruses to hijack the metabolism of their host cell(s) and manipulate them to produce viral progeny strengthens the need for a conceptual shift towards calling virus-infected cells ‘virocells’, as this will help emphasize their differences from uninfected cells (Forterre, 2011, 2013). Viral infections can change a microbe’s metabolic outputs, impacting the composition and quantity of their biosignatures in ways as profound as the organism’s own genome. Combined experimental and in silico studies of virus growth on bacterial hosts have shown how the physiological state of the host cell can be reflected in the timing and level of virus production, whereas virus production is intimately linked to the availability of resources for protein synthesis (Mahmoudabadi et al., 2017). Since nutrient availability in the host’s environment impacts its ability to produce viruses, the productivity of virus infection can provide a readout of the metabolic demands of the living host (You et al., 2002). Further, computational modelling suggests that viruses evolve to optimally use the finite metabolic energy resources of their host cells (Kim and Yin, 2004). Virus growth may also be linked to host physiology in ways not previously appreciated; studies of viruses that infect bacteria, eukarya and archaea revealed delay times for virus production that correlate with the doubling times of their host cells (Jin and Yin, 2021). Similar virus–host interactions could be at play in other systems, and we must be prepared to identify them.

Virus–host interactions and viral effects on microbial biosignatures can be evaluated through tracking stable isotopic signatures. Autotrophic microbial life preferentially incorporates the lighter isotope available for each biogenic element, a feature often used to identify ancient microbial life and sources of input materials in extant life. Yet the exact signatures of this process depend on which organisms are performing a given metabolic process, how many enzymatic reactions take place in relevant metabolic pathways and how the enzyme(s) work. For example, fractionation factors are different for denitrification via fungi versus bacteria (Ostrom and Ostrom, 2017), or for methanogenesis depending on the initial substrate and the organisms involved (Whiticar, 1999; Hornibrook et al., 2000; Penning et al., 2006). When a virus infects a microbial host, it redirects its metabolism, thus possibly impacting these isotopic values. Such impact could be more dramatic if a virus were to encode an auxiliary metabolic gene that has a different fractionation factor than the host version of that gene. For example, kinetic measurements of virus and host versions of the same enzyme have revealed that the virus enzyme had a significantly lower *k*_cat_/*K*_M_ value than the host enzyme (Thompson et al., 2011). Such viral influences can lead to large variations in isotopic signatures, leading to uncertainty when distinguishing between abiotic and biological processes and in the utility of a particular biosignature (Schwieterman et al., 2018). While complex and potentially difficult for distinguishing biotic from abiotic processes, isotopic fractionation has the potential to reveal fundamental characteristics of biosphere metabolism, including the impact and contribution of viruses.

Isotopic fractionation measurements have long been applied to the geologic record of Earth to infer characteristics of the metabolisms of past biospheres and changes over time (Johnson et al., 2021). The isotope history of marine ecosystems throughout Earth’s history is well-studied (Zerkle and Mikhail, 2017; Krissansen-Totton et al., 2015). However, the isotopic composition of icy environments through time on Earth is less well-characterized but may provide useful analogue environments applicable to other planetary bodies such as Mars (Havig and Hamilton, 2019). Distinguishing viral influences on both present isotopic signatures and on their variation over multiple timescales may therefore provide a reference for interpreting isotopic biosignatures of past life on other worlds. To better understand the role of viruses in biogeochemical cycling on Earth in order to interpret and model geochemical cycles elsewhere, we propose the following experimental directions.

#### Future directions

- Improve understanding of how different viruses hijack their host’s cellular machinery.
- Further explore the broad range of virus–host interactions and dynamics in various ecosystems.
- Quantitatively estimate the role and impact(s) of virocell metabolism on Earth’s different biogeochemical cycles.
- Improve understanding of the extent and distribution of viral impacts on biosignatures for majorEarth biogeochemical cycles, including the potential magnitude of such effects.
- Search for the existence of any generalizable biosignatures associated with viral metabolic reprogramming to reduce uncertainty associated with biosignatures for life detection.

### Impact of viruses on human space exploration

Human spaceflight continues to evolve, and many new technologies and tests are being developed for the safety of crew members and planetary protection for future human-landed missions. However, many studies exclude viruses, despite their threats (and potential benefits) to many types of cellular life. Thus, questions remain as to how viruses interact with other viruses and their hosts in space environments. For example, how do natural versus artificial environments drive changes in the virome and how are viruses impacted and shaped by their environment? As human spaceflight expands to sustained missions beyond low Earth orbit (LEO), nearly every aspect of the mission (including ECLSS, human health and performance, lander operations and extravehicular activities (EVA)) must consider the impact of virus–host interactions. Otherwise, the impacts may prove to be analogous to the current concern about volatile contamination on the ISS where current instrument capabilities and measurements are less sensitive or erroneous due to environmental conditions untested prior to flight (Regberg et al., 2022).

#### Viral response to spaceflight environments

Viral studies in microgravity have been very limited, with most microbiology investigations focused on bacteria. Those have shown a variety of species-specific and even strain-specific morphological and physiological responses to the low fluid shear force and lack of liquid media convection in microgravity (as reviewed in Diaz et al., 2019; Acres et al., 2021; Sharma and Curtis, 2022). Without externally applied forces, nearly everything in a microgravity environment can remain suspended or quiescent. Brownian motion dominates, thus reducing the ability for host cells to gather nutrients (Zea et al., 2016), and thereby influencing the size, structure and organization of these host cells. Additionally, the physical limitations of microgravity could potentially modify the infiltration capabilities of viruses. Further, the lack of buoyancy effects may also influence viral dispersal and host encounter rates in both the spacecraft cabin atmosphere and quiescent fluid systems. Another response to microgravity is aggregation (Zea et al., 2018; Domnin et al., 2022), which might limit the ability of viruses outside an aggregate to encounter a host surface or increase host encounter rate once one host in an aggregate is lysed. Even with external forces (fans, pumps, etc.) moving liquid and gas loops, it is still unknown how well viruses can adhere to encountered surfaces. Simulated microgravity has been associated with a thicker cell membrane envelope in *Escherichia coli* (Zea et al., 2018), which could inhibit the ability of viruses to infect hosts. In contrast, lower membrane integrity was observed in *Vibrio fischeri* (now known as *Aliivibrio fischeri*; Vroom et al., 2021), which could make hosts more susceptible to membrane-disrupting events.

The effects of microgravity on virus dynamics can be measured through environmental sequencing of surfaces aboard space stations and crewmember microbiomes, yet there is a dismaying lack of virus-centric metagenomic analyses (according to Mora et al., 2019, there has been a single study). Although there may be limitations in sampling size, frequency and depth of coverage due to the technical and logistical challenges of obtaining spacecraft samples, several data sets from the ISS are already available for reanalysis (Be et al., 2017; Singh et al., 2018; Urbaniak et al., 2018, 2022; Checinska Sielaff et al., 2019; Avila-Herrera et al., 2020). In future studies, the virus-to-host ratio for known virus–host pairs is one statistic that can be estimated, and its distribution explored spatially, temporally and in response to natural or experimental perturbations. Targeted and untargeted metagenomic sequencing of microgravity samples would have complementary strengths allowing for comprehensive analyses that include data from broad surveys at the species and genus taxonomic levels and to narrowly focused studies concerning specific strain and variant population dynamics. Viral dispersal dynamics, host adsorption, infection and progeny productivity in spaceflight environments are extremely understudied and deserve significant further work.

#### Environmental control and life support systems

**Figure 3.**
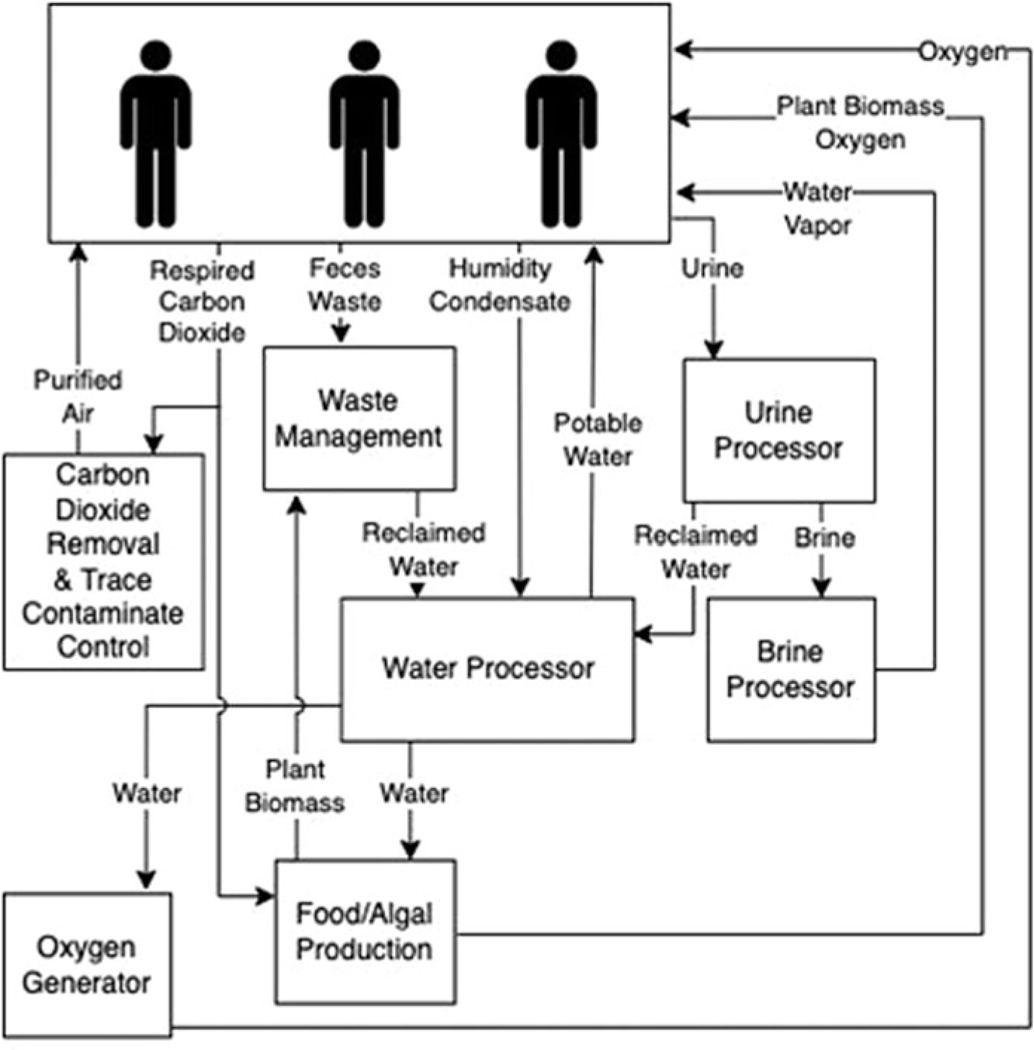
Review of spaceflight ECLSS potentially impacted by viruses. A simplified overview of environmentally controlled life-support systems for spacecraft and surface habitats that could be positively or negatively impacted by viruses.

ECLSS are imperative for supporting human spaceflight. These flight-proven technologies on the ISS control air composition and temperature, food and water and waste remediation. However, long-duration missions beyond LEO will require robust alternatives that do not rely on frequent resupply missions (Anderson et al., 2019). Closing the carbon loop through bioregenerative technologies is one approach for providing ECLSS. Algal photobioreactors can remove carbon dioxide (CO_2_), liberate O_2_, remove or alter waste and produce edible biomass (Matula and Nabity, 2019; Fahrion et al., 2021). These photobioreactors can withstand the dynamic temperature environment experienced within the ISS thermal control loops (Matula and Nabity, 2021; Matula et al., 2021). Preliminary spaceflight studies using algae for ECLSS observed thriving cultures (Helisch et al., 2020; Poughon et al., 2020). Likewise, extremophilic algae, lichen, cyanobacteria and fungi included in experiments mounted on the outside of the ISS and Space Shuttle survived weeks to months-long missions (de Vera et al., 2019; Malavasi et al., 2020). However, these studies did not characterize the full microbiome within the non-axenic cultures. This current lack is extremely important. For example, virophages can have profound implications for microbial nutrient cycling, often referred to as the microbial loop. Predator–prey simulation models indicate that the presence of virophages regulates helper virus and algal host dynamics altering carbon flux through the microbial loop in aquatic ecosystems (Yau et al., 2011). To survive unfavourable conditions (e.g. microgravity, viral infection and antimicrobials), some microbes can form protective barriers such as biofilms. Bacteria exude a variety of organic matter dubbed extracellular polymeric substances, which forms the biofilm and acts like glue securing and protecting the bacteria allowing them to remain hydrated, control local pH and other services. Biofilms can benefit certain systems such as the rhizosphere (plants root zones) aiding in food production. However, biofilms can also be detrimental by corroding or clogging machinery that supports ECLSS, or preventing wound healing in skin abrasions, to name just a couple of examples (Landry et al., 2020). Recently, highlighted in Justiniano et al. (2023) are the knowledge gaps in our understanding of microbiomes (including viruses) and biofilm formation in space, although we do know that viruses can adapt new strategies to prevent bacterial growth or kill organisms within biofilms. Understanding potential biome or virome shifts within these systems over long durations may elucidate the need for specific algal species selection, causes of viral or bacterial-based system failures and operational considerations (Fig. 3; Matula and Nabity, 2019).

#### Bioregenerative ECLSS

Efforts on the ISS have focused on plants for CO_2_ removal and O_2_ production, as a source of multivitamins, for urine reuse and to promote psychological well-being (Dzhos et al., 2021). On Earth, plant viruses can be a threat to agriculture, and disease management relies on rapid and accurate viral identification (Rubio et al., 2020). Many plants have been grown under microgravity, including flower-producing species, herbs and vegetables. However, due to the stress of spaceflight, viral infection of these plants can be hugely influential on functionality. A recent study found that spaceflight factors significantly affect tomato plants (Dzhos et al., 2021), increasing the productivity of the plants and the concentration of some vitamins such as carotenoids, which are important for crew members on long-term space missions. Importantly, plants grown from seeds that were in space for half a year prior to germination were resistant to viral infection. Moreover, seed resilience of many plant species (amongst other organisms) was tested by keeping them outside the ISS for 558 or 682 days, thus exposing them to high levels of radiation (Tepfer and Leach, 2017). All seeds were able to germinate after the shorter flight, but only seeds with a stronger coat could survive the longer radiation exposure. While these studies show promise for cultivating plants on long-term voyages, our understanding of plant–virus interactions on short flights is extremely limited and we have no information for long-term flights.

#### Human physiology and the viral ecology of crewed spacecraft

In space-bound crewmembers, latent viruses are shown to reactivate more than in matched controls. Sometimes this reactivation occurs before leaving Earth and is presumably stress related. However, there are several plausible reactivation triggers associated with spaceflight itself, such as ultraviolet or ionizing radiation (Pavletíc et al., 2022), but precisely which mechanisms might be responsible for increased virus reactivation in crewmembers have not been determined. Reactivation has included herpes simplex virus, Epstein–Barr virus and varicella zoster virus (Stowe et al., 2000; Mehta et al., 2004; Pierson et al., 2005; Rooney et al., 2019). After 6–12 months in space, crewmembers experienced significant changes in their microbiome (both internal and external) that led to rashes and hypersensitivity episodes (Voorhies et al., 2019). These symptoms warrant further investigation into the effects of long-term space environment exposure on humans combined with their accompanying microbes and viruses.

The community composition of viruses and other organisms on the ISS is mostly comprised of viruses that only infect non-human cells, such as bacteriophages. This composition differs from that of terrestrial space analogues (e.g. MDRS and HI-SEAS), thus pointing towards the space environment as the likely trigger (Mora et al., 2019). Although these viruses do not infect humans, they can be transported back to Earth and released into Earth environments via plants, humans and other entities that can harbour them. Continued efforts are needed for quarantining people within space stations for long-term space travel and increased screening for crewmembers returning to Earth. A viable way to test viral transmission and educate humans, especially those preparing for space travel is through simulations that consider epidemiology parameters (Patel et al., 2021). Although it might be tempting to contemplate elimination of viruses from spacecraft wherever possible, we should be sensitive to the unanticipated positive roles viruses may play and the unreasonable resources that viral elimination would entail. As well, a virus’s presence can be beneficial by sometimes warding off more pathogenic relatives by cross-protection or superinfection exclusion (Folimonova et al., 2020). Immunologic ‘eustress’ can promote a healthy organism, as is seen by comparison to the physiological deficits of germ-free animals (Round and Mazmanian, 2009). Indeed, in some cases, viruses can be symbiotic with their host organisms (Roosinck, 2011). As such, viruses are key players in crewmember health and safety and in the ecology of crewed environments.

#### Spaceflight operational impacts

Given the differences in viral dispersal patterns observed in low-gravity environments and the extreme conditions under which viruses persist and interact with hosts (see Section ‘Viral response to spaceflight environments’), even surfaces often considered sterile, or at least clean, must be investigated. Some bacterial species – for example, *Bacillus pumilis* strain SAFR-032 (Tirumalai et al., 2013) – are refractory to pre-flight sterilization, and some could be tolerant as well given the extreme diversity of viral morphotypes (including lipid-free viruses, see Fig. 1). Even with perfect sterilization of equipment (which is essentially unobtainable), it is not currently possible to achieve a virus-free environment on a manned space mission because of post-launch viral shedding by crewmembers (*vide supra*). The frequency of sampling, maintaining and cleaning life support systems will impact day-to-day spaceflight operations, during both cruise phase and orbital or surface sustained missions. EVA and associated planetary protection protocols must also be accommodated: for example, protecting areas with high-scientific value from contamination (e.g. Rummel et al., 2014). Modifications of operations may entail landing further from a planetary surface sample site, designing new vehicles, requiring more stringent cleaning protocols or considering entirely new sample sites (e.g. Meyer et al., 2019). Additionally, contamination restrictions during EVAwill narrow the design of space suit systems (Willson et al., 2014). Attempts to dictate planetary protection protocols without fully understanding viral dispersal and survivability will potentially result in overly restrictive constraints that ultimately hinder scientific discovery or alternatively inadequate protocols that do not achieve proper containment.

To understand the impact of viruses on human space exploration, we suggest that the following experiments be prioritized.

#### Future directions

- Characterize differences in dispersal, aggregation and adsorption processes of viruses in both fluid and air microgravity environments.
- Compare the ability of viruses to adsorb to and infect hosts during and after microgravity-induced cellular changes.
- Investigate virus–host interactions, especially in response to perturbation effects on virus-to-host ratios.
- Develop enhanced capabilities for onboard biosurveillance.
- Investigate viromes in built environments relevant to space, including the ISS.
- Explore virus–host dynamics for both human- and plant-associated microbiomes in space.
- Assess space-associated triggers that cause latent viral infections to become virulent.
- Quantitatively estimate the role and impact(s) of virocell metabolism in spaceflight environments.
- Identify viral loading impacts significant to both planetary protection requirements, and preservation of human health. Explore desirable modifications to spaceflight operations (travel, landing and EVA) that could ameliorate such loading impacts.
- Characterize viral influence on microbial biofilm composition and growth dynamics in human, algal and plant systems under various space-related conditions.

### Detection of viruses on Earth and elsewhere

A variety of instrumentation exists that can detect both microbial biomarkers and viruses. These types of techniques have often been used on Earth to study viruses (Sommers et al., 2021; Vincent and Vardi, 2023). However, these same techniques can be difficult to conduct with automated systems in space and have yet to be designed specifically for space missions. Therefore, it is currently unclear if and how informational molecules can be reliably detected in environmental samples beyond Earth. Engineering investigations must be paired with science goals to further develop innovative, flight-ready technologies to detect and characterize viruses, virus-like particles and virus genomes efficiently and accurately. Once developed, such technologies could then be applied in Earth analogue systems to test performance and limitations for detecting viruses and virus-like particles. Additionally, these instruments and protocols can be incorporated into standard operating procedures for planetary sample missions.

While autonomous virus-detection technology is not yet ready, the future is promising. For example, solid-state nanopore-based biosensors are currently being designed for spaceflight and have proven capability for evaluating different biomarkers (i.e. DNA/RNA, proteins and whole viral particles – spanning from a few nanometres to over 100 nanometres in diameter). Solid-state nanopore sensors would be particularly useful in space missions because they also can measure particle-size distributions within virus populations and discriminate between viral particles by monitoring the change in electrical current as particles pass through an electrically biased pore or by measuring the mass density of viruses (Zhou et al., 2011; Arjmandi et al., 2014; McMullen et al., 2014; Akpinar and Yin, 2015; Wu et al., 2016). Researchers have adapted flow cytometry to the scale of virus particles; specifically, flow virometry employs more powerful lasers, reduced diameter fluid flows, wider-angle sampling of scattered light and fluorescent labelling of particles (Bhat et al., 2022). Additionally, microscopy has been used to evaluate microbial and viral populations on Earth. Microscopy technologies paired with spectroscopic techniques, can inform the physical shapes and boundaries belonging to certain chemical or mineralogic compositions (Zhang et al., 2013). While an atomic force microscope was used during the Phoenix Mars Lander to investigate Martian soils (Pike et al., 2011) and recently, a scanning electron microscope was added to the ISS (Own et al., 2018) higher performance complex space-qualified microscopes have yet to be developed that can reliably detect virus-like particles. Another technique that could be applied to space virology is NanoSIMS, which performs in situ quantitative trace sample analysis with exceptional sensitivity and spatial resolution (Mayali, 2020; Pett-Ridge and Weber, 2022). NanoSIMS has been used to detect viruses (Gates et al., 2018) and map their elemental and isotopic distributions in complex communities and in mineralized samples (Pacton et al., 2014). Also, the Network for Life Detection is currently using NanoSIMS to discern between abiotic and biological signatures based on the differences in elemental and isotopic patterns of cellular organisms versus minerals and other precipitates. These technologies complement spectroscopic methods by providing orthogonal evidence for viral and non-viral life (Zhang et al., 2013). Overall, there are multiple instrument designs either waiting to be developed or currently being developed for detecting viruses and life beyond Earth.

Environmental virology techniques could also complement life detection instrumentation. In conjunction with other previously described instruments, high fidelity sequencing methods and high-resolution structure analysis could also confirm the presence of viruses on other worlds (Bamford et al., 2005). Moreover, to reduce signal-to-noise problems, relic or environmental DNA can be removed from a system using propidium monoazide to enhance detectability of intact viruses and microbes (Wagner et al., 2008; Weinmaier et al., 2015). Thus, methods of environmental virology could also be necessary for life detection and returned samples (Janjic, 2018; Ricciardi et al., 2022; Table 3). Much work remains to integrate both engineering and science perspectives. While some instrumentation, such as light, electron and atomic force microscopy, has been used on Mars and in space on the ISS (Pike et al., 2011; Own et al., 2018), instrumentation in development must also be tested in extreme analogue environments to explore and overcome limitations. Moreover, for planetary protection purposes, utilizing instrumentation for detecting viruses should be incorporated into standard operating procedures for flight and sample return missions. To fully understand the presence of viruses in space environments, new technologies need to be developed and include the following.

#### Future directions

- Develop innovative autonomous flight-ready technologies for efficient and accurate detection and characterization of virus genomes.
- Apply such technologies to Earth analogue systems to explore prospects and limitations for detecting viruses and virus-like particles.
- Incorporate measurements of viruses in standard operating procedures for sample return missions and subsequent examinations.

## Conclusion

For effective pursuit of astrobiology questions, it is critical to understand how life on Earth functions, as it is currently the only place where life is confirmed to exist. Viruses are key contributors to Earth’s ecosystems; however, much remains unknown regarding their influence on cellular life, role in evolutionary history and their fundamental physical interactions with the Earth system. Basic ecological factors, such as persistence and decay under various scenarios, also remain underexplored. Likewise, safe and effective crewed deep space travel will require a thorough understanding of the human microbiome in space, including viruses. Here, we have outlined ways in which focused astrovirological investigations can broadly advance the goals of space science. Across the diverse disciplines that make up astrobiology and space biology, we highlight several classes of investigations which cannot afford to neglect viruses. Most of the diversity of life on Earth is comprised of viruses, whether diversity is defined by number of species, type of genetic information, number of individuals (Breitbart et al., 2002; Roossinck, 2012; Paez-Espino et al., 2016; Parikka et al., 2017; Mushegian, 2020), absence of any universally present gene, mode of replication or number of unique (i.e. non-homologous) genes (Koonin and Wolf, 2012). Viruses should be explicitly considered in organism-level astrobiological investigations. The ability to detect virus-like entities must be a point of assessment for instruments and missions to directly detect extraterrestrial organisms for planetary protection, human spaceflight safety and sample return (Janjic, 2018; Ricciardi et al., 2022). When organisms are used as model systems, viruses should be adequately represented to reduce biases and increase utility of diverse astrobiological studies.

‘Whether viruses are alive or not may be a moot question, but if a virion (or virus-like particle) were to be unequivocally detected in an extraterrestrial sample, very few people would claim that this would not be evidence for life – wherever that sample was from’.

– Berliner et al. (2018)

## Figures and Tables

**Table 1.** Comparison of cellular and non-cellular entities—cells possess each of these four significant genetic and structural materials

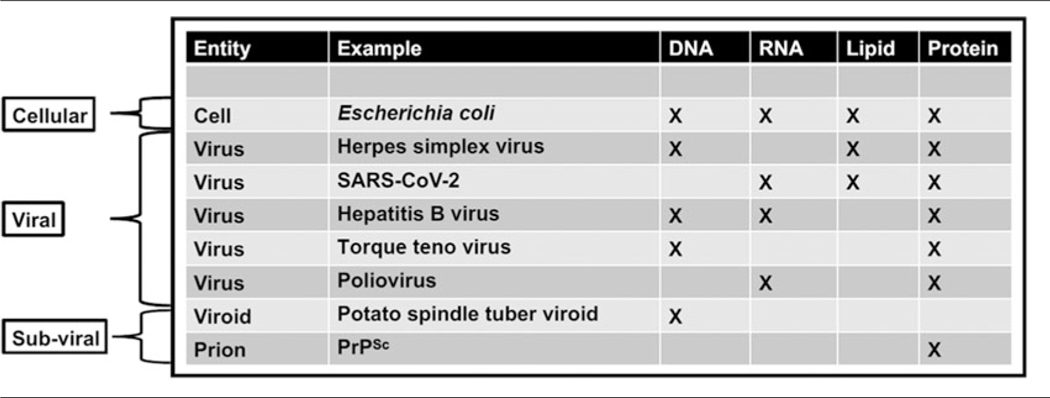

In the case of viruses and subviral elements, only reduced suites of these key molecules are present. Since non-cellular entities can possess either DNA or non-DNA genomes and may lack lipids, the search for extraterrestrial DNA, RNA or lipid alone would fail to detect some of these entities.

**Table 2.** Example locations where viruses have been observed through isolation or genomic studies

Location	Condition	Reference
Atmosphere		
Droplets suspended in the atmosphere	N/A	Reche et al. (2018)
Air samples (Korea)	~−4 to 20°C, ~4–15mb vapour pressure	Whon et al. (2012)
Freshwater sites		
Lakes, rivers, wetlands (Ontario, Canada)	3.5–18°C, pH 5.85–9.09	Kyle and Ferris (2013)
Lake Ontario, Lake Erie (Great Lakes)	N/A	Mohiuddin and Schellhom (2015)
Acid mine drainages		
Mine sites (Southern China)	N/A	Gao et al. (2022)
Sudbury Igneous Complex (Ontario, Canada)	14°C, pH 2.45–4.00	Kyle and Ferris (2013)
Permafrost-associated soils		
Alaska Peatland Experiment (APEX) site	− 1.5°C	Trubl et al. (2021)
Stordalen Mire field site near Abisko, Sweden	0–17°C, pH 3.4–6.2	Trubl et al. (2018, 2019)
Permafrost thaw gradient (Sweden)	0–17°C, pH 3.4–6.2	Emerson et al. (2018)
Ice cores or cryoconite holes		
Canadian Arctic ice core	−31 to −9°C, 4–6 psu salinity	Wells and Deming (2006a)
Arctic nepheloid layer	−12 to 8°C, 50 psu salinity, 200 atm	Wells and Deming (2006b)
Cryopeg brine, sea-ice brine, melted sea ice	N/A	Zhong et al. (2020)
Antarctic cryoconite holes	N/A	Sommers et al. (2019)
Soda Lakes/solar salterns		
Mono Lake (CA, USA)	~5–14°C, pH ~ 10, 70–85 psu salinity	Brum and Steward (2010)
Solar salterns and salt lakes (Sicily, Italy, Thailand, Israel, Slovenia, Spain)	Viruses grown under 37°C, pH 7.2, 30% w/v salt water	Atanasova et al. (2012)
Magadi and Shala Lakes	Viruses grown under 37°C, pH 9, 5% NaCl	van Zyl et al. (2016)
Great Salt Plains National Wildlife Refuge (OK, USA)	Virus grown under 15–30°C, pH 6–9, 5–20% NaCl	Seaman and Day (2007)
Hot springs		
Obama hot spring sediment (Japan)	70–90°C, pH 6–9, 0–5.8% NaCl	Nagayoshi et al. (2016)
Hot Springs (Pozzuoi, Italy)	87–93°C, pH 1.5	Haring et al. (2005)
Yellowstone hot springs (USA)	Bear paw (74°C, pH 7.34); octopus (93°C, pH 8.14)	Schoenfeld et al. (2008)
Fumarole		
Campi Flegrei volcano (Pozzuoli, Italy)	81–96°C; pH 1–7	Baquero et al. (2020)
Deep-sea sediments		
Deep subsurface sediments of the Peru margin	~318 mbsf	Engelhardt et al. (2013)
Subseafloor sediment continental shelf of Peru	5–159 mbsf	Engelhardt et al. (2015)
Baltic Sea subseafloor sediments	37.1–437.1 m depth below water	Cai et al. (2019)
Deep-sea hydrothermal vents		
Wocan and Tianxiu hydrothermal fields	N/A	Cheng et al. (2022)
Guaymas Basin, Gulf of CA	2000 m depth below water; virus isolate grown under 80°C, pH 6.3, 2% NaCl	Thiroux et al. (2021)
Other		
Chemically harsh conditions	Virus survival in 5–9 M urea	Gupta et al. (1995)
Chemically harsh conditions	Virus survival in 99% acetonitrile	Olofsson et al. (2001)
Ice cubes	N/A	Jalava et al. (2019)

This table provides examples, and it is not a comprehensive review of all locations where viruses have been observed/isolated. psu, practical salinity unit; mbsf, metres below sea floor.

**Table 3.** Possible virus detection methods: how can we detect extraterrestrial viruses?

Virus detection method	Advantages	Challenges
Infection	Highly specific for virus identification and host response	1. Requires correct host 2. Requires suitable conditions
Virion detection	Unique structures enable direct detection	1. Virions may not represent active viruses 2. Could produce false positives
Metagenome sequencing	Very broad net theoretically allowing any virus to be identified	1. Sequences may not resemble known viruses 2. Nucleic acid may be hard to purify or amplify 3. Exoviruses may not contain nucleic acids
Virus effects on hosts	May be detectable remotely	1. May be difficult to differentiate between infected and uninfected hosts 2. No detectable hosts In environment
Microscopy indicators (SEM, TEM, AFM)	Provide potential indication of viruses to be confirmed by methods above	1. Typical virion size is small 2. Very high resolution is necessary 3. Morphologies may be indistinct

Of the currently known methods for virus detection, those suitable for astrobiology applications are challenging to imagine but we can leverage off the suite of methods already in use on Earth.
